# Light irradiation for treatment of acute carbon monoxide poisoning: an experimental study

**DOI:** 10.1186/s40560-016-0181-0

**Published:** 2016-09-02

**Authors:** Taku Tanaka, Takeshi Kashimura, Marii Ise, Brandon D. Lohman, Yasuhiko Taira

**Affiliations:** 1 Medical Emergency and Disaster Center, Kawasaki Municipal Tama Hospital, 1-30-37 Shukugawara, Tama-ku, Kawasaki, Kanagawa 214-8525 Japan; 2 Emergency and Critical Care Medicine, St. Marianna University School of Medicine, 2-16-1 Sugao, Miyamae-ku, Kawasaki, Kanagawa 216-8511 Japan

## Abstract

**Background:**

Because treatment modalities for carbon monoxide (CO) poisoning, especially normobaric oxygen and hyperbaric oxygen therapies, have limited effects and hyperbaric oxygen is not available at the scene where treatment is most needed, we conducted a study to determine and compare rates of carboxyhemoglobin (COHb) dissociation achieved in human in vitro blood samples under light radiation emitted at three levels of illuminance. This was done with a view toward eventual on-site application.

**Methods:**

We drew blood from 10 volunteers, prepared 10 red blood cell solutions, and subjected each solution to a CO bubbling procedure to increase the COHb saturation. Samples of each bubbled solution were then divided between 3 beakers (beakers A, B, and C) for a total of 30 beakers. The solution in each beaker was exposed to a continuous flow of oxygen at 50 mL/min, and simultaneously for a period of 15 min, the beaker A and B solutions were irradiated with light emitted at 500,000 and 100,000 lux, respectively, from a halogen light source. The beaker C solutions were exposed to room light. At 3, 6, 9, 12, and 15 min, a 50-μL sample was pipetted from each of the 30 beakers for determination of its light absorbance and the COHb dissociation rate.

**Results:**

Under each of the experimental conditions, dissociation progressed but at different rates, and starting at 3 min, the differences in rates between conditions were significant (*P* < 0.01). The dissociation rate was greatest with light emitted at 500,000 lux.

**Conclusions:**

Our results point toward the possibility of readily performed, acute photodissociation therapy for patients with CO poisoning.

## Background

Awareness of the deadly effects of carbon monoxide (CO) dates back to the time of the ancient Greeks and Romans, when the gas was used for executions [1]. To this day, CO poisoning remains one of the most common types of poisoning, accounting for more than 60 % of poisoning deaths in Japan, accidental or otherwise [2]. CO is a tasteless, odorless, and non-irritating but highly toxic gas that confers significant long-term morbidity and is often associated with severe delayed neuropathology [3]. Despite the aggressive treatment strategies available, morbidity and mortality rates attributable to CO poisoning remain high [4].

Because of the intrinsic properties of CO and lack of specific clinical features of CO poisoning, the condition is difficult to detect, and it can mimic other common disorders. Therefore, the true incidence of CO poisoning is unknown, and it is likely that many cases go unrecognized. CO is a by-product of the incomplete combustion of hydrocarbons, and it is commonly produced by domestic heating appliances and systems, charcoal burning, fires, and car exhaust. CO poisoning accounts for many deaths by suicide.

CO toxicity results from the formation of a carbon-hemoglobin complex, carboxyhemoglobin (COHb), which is over 200-fold more stable than oxyhemoglobin (O_2_Hb) [5, 6]. An increased blood COHb concentration will compromise oxygen transport by hemoglobin and thus decrease oxygen delivery to vital organs and tissues. Furthermore, not only does COHb have a long half-life, but at a 20 % concentration, more than 8 h may be required for CO to fully dissociate from the complexed hemoglobin [7]. The importance of effectively eliminating the toxic effect of CO cannot be overemphasized. However, present treatment modalities, especially hyperbaric oxygen (HBO) and normobaric oxygen (NBO) therapies, have limited effects, and HBO therapy especially remains controversial and not readily available at the scene where treatment is most needed. Phototherapy, specifically photodissociation of COHb, appears to be a viable alternative because there are no known complications. With a view toward use in clinical cases of CO poisoning, we conducted a study to determine and compare rates of COHb dissociation achieved in human in vitro blood samples under light radiation emitted from two different light sources at three different levels of illuminance.

## Methods

### Blood samples

**Fig. 1:**
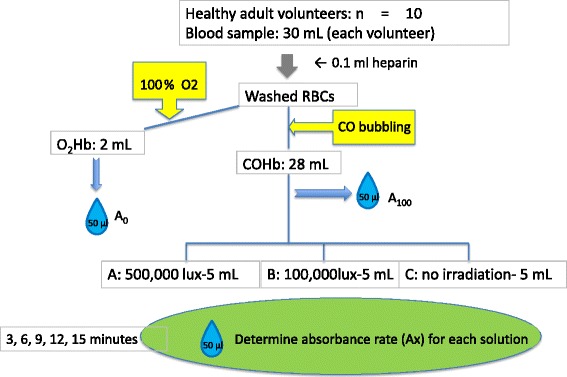
Flow diagram showing preparation of blood samples for exposure to the three different experimental conditions and the subsequent determination of absorbance rates

Under approval granted by the Ethics Committee of St. Marianna University School of Medicine, Japan, we drew blood from 10 adult volunteers (smokers *n* = 2, non-smokers *n* = 8) recruited from our staff. The volunteers ranged in age from 28 to 61 years, with a mean of 37.9 years. Thirty milliliters of blood was collected from each volunteer by direct venipuncture of the brachial vein, immediately anticoagulated with 0.1 mL heparin (Novoheparin, Mochida Seiyaku, Tokyo, Japan), and then washed. The specimen was then centrifuged at 3000 rpm for 10 min, the plasma supernatant was removed, the erythrocytes were re-suspended in a 9-g/L normal saline solution, and the suspension was centrifuged. This process was repeated three times before the tube was inverted two or three times until the contents were mixed to the point of turbidity. Next, 2 mL of the red blood cell solution was pipetted out of the tube and exposed continuously to 100 % pure oxygen at 60 mL/min to produce 100 % O_2_-saturated hemoglobin (100 % O_2_Hb) (Fig. 1).

### CO bubbling

**Fig. 2:**
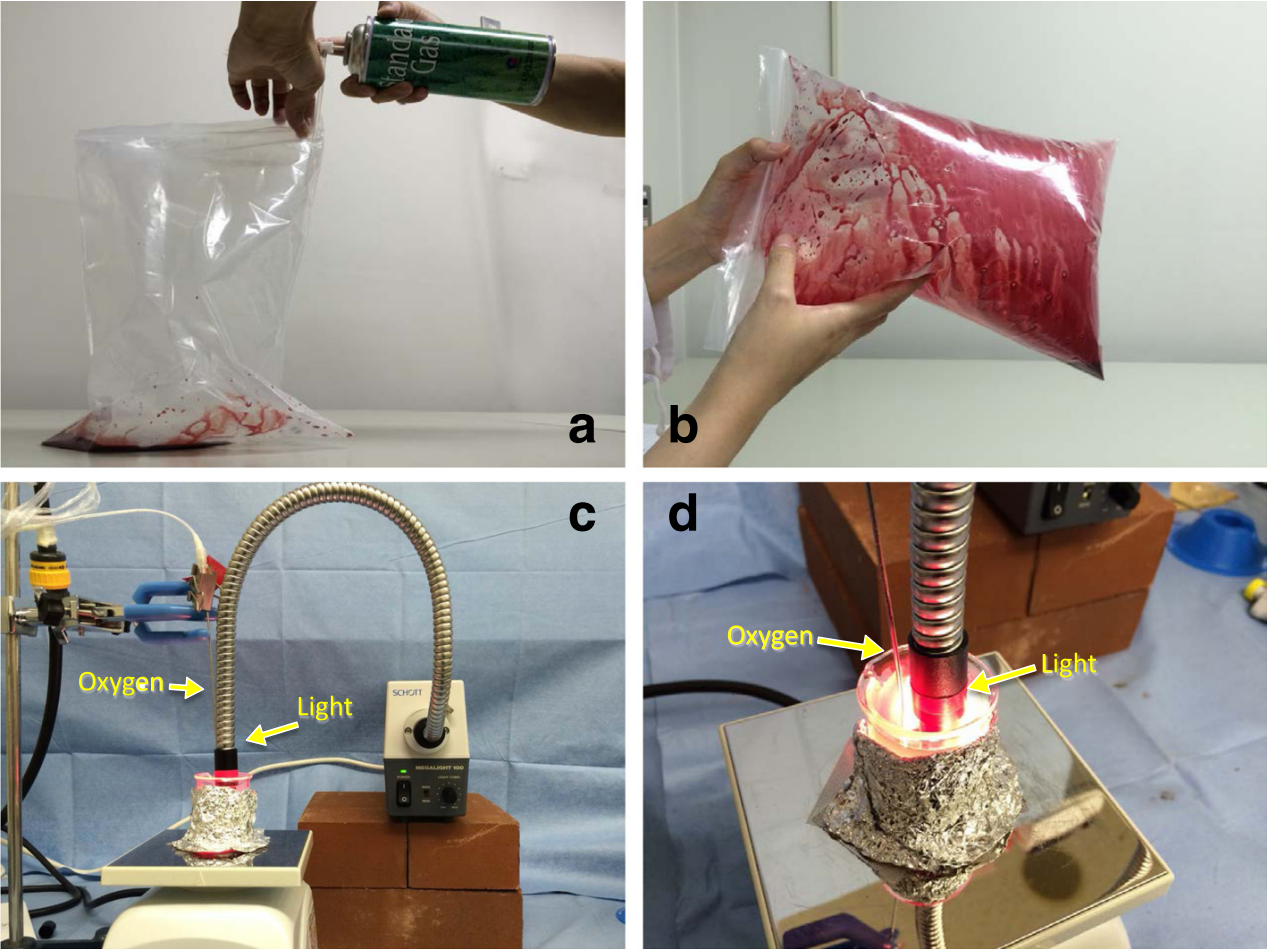
CO bubbling. **a** CO is injected into a Ziploc bag containing the red blood cell solution until the bag is fully inflated. **b** The inflated bag is then vigorously shaken for at least 60 min, resulting in 90 % COHb production. **c**, **d** The sample solution, now in a beaker, is simultaneously exposed to light (500,000 lux, 300,000 lux, or simply room light) and to a continuous flow of oxygen at 50 mL/min. The oxygen is delivered via a copper nozzle connected to an oxygen gas cylinder

The remaining 28 mL of the red blood cell solution was prepared for CO bubbling, a procedure that is designed to increase the COHb saturation. The procedure consists of the following series of steps: (1) the red blood cell solution is placed at the bottom of a Ziploc freezer bag, and a flexible, plastic spray nozzle belonging to a CO canister (GL-Sciences, Ltd., Tokyo, Japan) is positioned at the mouth of the bag; (2) the air is squeezed out of the bag, which is then sealed by hand, and to avoid any tearing, the sealed bag is placed in a second Ziploc bag, which is also sealed; (3) the nozzle is attached to the CO canister, and CO is injected into the bag until it is fully inflated (Fig. 2a); and (4) the CO-inflated bag is then vigorously shaken for at least 60 min, which we know will result in over 90 % COHb production (Fig. 2b).

After the CO bubbling procedure, three separate 5-mL samples of the solution were collected from the bag and placed into three separate 20-mL beakers: beaker A, beaker B, and beaker C. In all, 30 samples (3 samples × 10 volunteers) were obtained, and each was placed in its own beaker. A halogen light source (MegaLight 100, SHOTT-MORITEX, Tokyo, Japan) attached to a 25-cm-long flexible stainless steel gooseneck light guide was placed 10 mm above the surface of each sample. The light emitting end of the light guide was 6 mm in diameter. We administered a continuous flow of oxygen at 50 mL/min by plunging a copper nozzle (length 100 mm, diameter 4 mm) connected to an oxygen gas cylinder into the red blood cell solution, and at the same time, for a period of 15 min, we irradiated the beaker A blood sample with light at a total luminance of 500,000 lux and the beaker B sample with light at a total luminance of 100,000 lux. The beaker C sample was not irradiated but rather was left exposed to room light (Fig. 2c, d). At 3-min intervals (3, 6, 9, 12, and 15 min), a 50-μL sample was pipetted out of each beaker for determination of its light absorbance and its CO dissociation rate.

A distance of 10 mm between the light guide tip and the red blood cell surface is the optimal distance at which the flexible light guide can safely emit light without causing any blood splatter from the continuous oxygen administration. Under the optical illumination with 500,000 lux maintained for 15 min, the temperature of the blood in the beakers was below 32 °C, and therefore, hemoglobin protein denaturation was not a concern during the irradiation process. In addition, each beaker was firmly sealed with aluminum foil to prevent the escape of any irradiation light. The light reaches the bottom of the beaker and there measures 50–60 lux.

### Measurement of the photodissociation rate

**Fig. 3:**
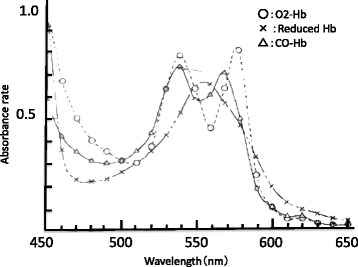
O_2_Hb, reduced O_2_Hb, and COHb waveforms

It is well established that O_2_Hb and COHb each has its own specific light absorption spectra that are characterized by a bimodal curve, each with two maximal spectrophotometrically determined absorption peaks: 540 and 576 nm for O_2_Hb and 538 and 568 nm for COHb. The absorption spectra may be altered by varying the ratio between the O_2_Hb and COHb concentrations, and the morphology of the resulting waveforms will be by modified accordingly. It is also known that a small amount of sodium hydrosulfite can reduce O_2_Hb, thereby altering its absorption curve from a bimodal to a monomodal wavelength with an absorption peak of 555 nm. (Fig. 3) [8, 9].

Because the aim of this study was to determine the rate of COHb dissociation resulting from exposure to light radiation, we selected a reduced O_2_Hb monomodal curve absorption value of 555 nm and an absorption value of 538 nm from the COHb bimodal curve. To this end, we removed two samples of 50 μL each, one from the previously prepared sample containing 100 % O_2_Hb, and one from the CO-bubbling bag, and we placed these samples in two separate test tubes before adding to each of them 10 mL 0.1 % sodium carbonate and then 0.0275 g (1 micro-spoon) sodium hydrosulfite, and we gently shook the samples for a few seconds. After allowing the solution to settle for 15 min, the samples were analyzed by spectrophotometry (BioSpectrometer Basic, Eppendorf, Hamburg, Germany) so that we could determine the parameters necessary to tabulate the O_2_Hb (*A*_0_) and COHb (*A*_100_) constants that would be needed to calculate the COHb dissociation rate.

A 50-μL sample was then collected every 3 min over a period of 15 min (at 3, 6, 9, 12 and 15 min) from beaker A, beaker B, and beaker C, and these samples were subjected to the same buffering procedure described above. Subsequently, all collected samples were subjected to spectrophotometry in which the constant values were used to determine the light absorbance spectrum (*A*_*x*_) of each sample.

### Calculating CO saturation

For all samples, a bimodal curve was projected on a Cartesian coordinate system visible on the spectrophotometer screen. The abscissa and ordinate axes represent the wavelength and absorbance spectra, respectively. From the given E555 and E568 wavelengths on the *x* axis, we were able to identify the corresponding absorbance values on the *y* axis for O_2_Hb and COHb in all samples tested.

Next, the respective constant values of *A*_0_ and *A*_100_ for both O_2_Hb and COHb and the *A*_*x*_ values for the three samples were calculated as follows: *A*_0_ = E538/E555, *A*_100_ = E538/E555, and *A*_*x*_ = E538/E555, which were then inserted and tabulated according to the following COHb concentration equation: COHb (%) = (*A*_*x*_ − *A*_0_) / (*A*_100_ − *A*_0_) * [9]. The value obtained is the dissociation rate, and the rate was calculated for each of the 30 samples obtained at each of the various time points.

### Statistical analysis

For each of the 10 samples obtained at each time point, the mean ± SE COHb dissociation rate was calculated. The rates were then plotted per condition (i.e., per beaker A, B, or C), per time point. Differences in the mean ± SE dissociation rate between the three conditions were analyzed by repeated-measures ANOVA and Tukey’s HSD test. Correlation between COHb saturation (shown as a percentage) and the rate of change was tested by Pearson’s correlation coefficient. All statistical analyses were performed with Stat Flex ver. 6 (Artech Co., Ltd., Osaka, Japan), and *P* < 0.05 was considered significant.

## Results

**Fig. 4:**
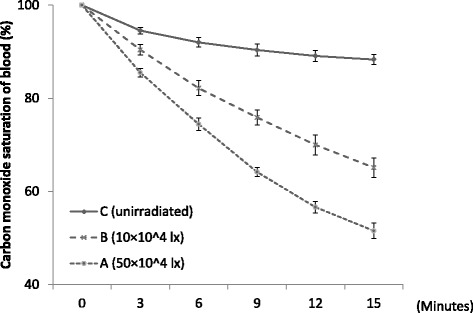
COHb dissociation curves plotted from mean ± SE dissociation rates for the total solution samples under the three different light exposure conditions over a 15-min time period

**Fig. 5:**
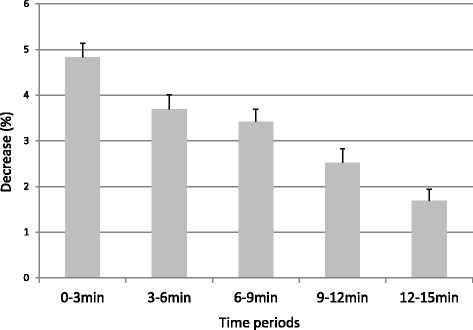
Bar graph of the dissociation rate for each of the 3-minute time periods in samples exposed to light at 500,000 lux

**Fig. 6:**
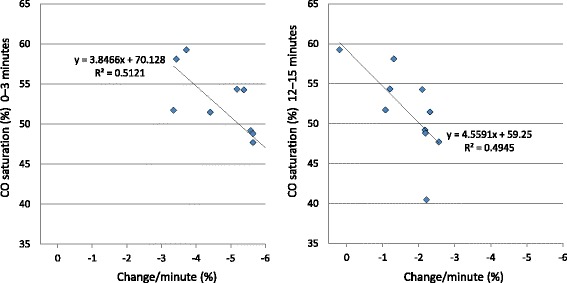
Correlation between CO saturation and the change in the dissociation rate during the 0–3-min and 12–15-min time periods

The study subjects, pertinent subject characteristics, and individual study data are shown in Table 1. We drew COHb dissociation curves (Fig. 4), and in comparing these curves, we found that the dissociation rate determined for the beaker B (100,000 lux) samples was higher than that for the beaker C (no irradiation) samples but lower than that for the beaker A (500,000 lux) samples. Under each of the experimental conditions, dissociation progressed at different rates, but starting at 3 min, the differences in rates between conditions were significant (*P* < 0.01). Under exposure to light at 500,000 lux, the dissociation rate decreased uniformly over time with no noticeable difference between the time periods (Fig. 5). Correlation between COHb saturation and the change in the dissociation rate was significant for the 0–3-min time period and the 12–15-min time period at *P* = 0.020 and *P* = 0.023, respectively (Fig. 6). Tukey’s multiple comparisons analysis confirmed all statistical differences that we found (*P* < 0.01).

**Table 1:** Study subjects, pertinent subject characteristics, and individual study data

Subject	Age (years)	Sex	Smoking	500,000 lux		100,000 lux		No irradiation		Change rate
				3 min	15 min	3 min	15 min	3 min	15 min	0–3 min
1	44	M	No	83.9	54.3	87.9	62.0	95.1	91.5	−5.38
2	41	F	No	82.0	40.5	83.4	47.8	93.8	87.5	−6.02
3	28	F	No	83.1	47.7	97.9	70.9	97.8	90.3	−5.64
4	42	M	No	89.7	58.1	92.1	69.9	89.7	93.3	−3.43
5	40	F	No	83.3	49.2	92.4	71.6	98.6	91.1	−5.58
6	30	M	No	83.1	48.8	92.4	62.2	95.2	82.8	−5.64
7	32	M	No	84.5	54.3	89.4	69.6	91.4	84.7	−5.18
8	30	M	No	88.8	59.2	90.4	68.3	93.6	85.9	−3.72
9	61	M	Yes	90.0	51.7	89.9	64.9	94.4	90.4	−3.35
10	31	M	Yes	86.8	51.5	88.3	63.8	91.3	85.8	−4.41
Mean	37.9	–	–	85.5	51.5	90.4	65.1	94.5	88.3	−4.84

Two of the volunteers were chronic heavy smokers (subject 8: 2 packs/day for >30 years and subject 9: 1 pack/day for <10 years). Although the study group was too small for meaningful comparison between smokers and non-smokers, we noted that there was no significant difference in dissociation rates between them.

## Discussion

An estimated 58,000 emergency cases of CO poisoning occur yearly in Japan, and the resulting estimated cost of over 1 trillion 75 billion yen (8,743,000,000 USD) per year [10] translates to a devastating socio-economic impact. The symptoms of CO poisoning are non-specific [11] and usually manifest when the CO concentration rises above 10 % [12]. However, the reported association between a patient’s blood level and that patient’s clinical condition is poor [11, 12]. The general consensus is that levels below 60 % do not result in coma or death [5]. In non-smokers, the average CO concentration is 1 %, whereas in heavy smokers, the average concentration may reach 15 % [13]. CO causes hypoxia by combining with hemoglobin in the blood to form COHb and shifting the O_2_Hb dissociation curve to the left [13]. Its affinity for hemoglobin is more than 200 times that of oxygen [14], and it easily displaces oxygen from hemoglobin, whereas COHb liberates CO very slowly, resulting in the formation of COHb with even negligible amounts of inhaled CO.

A small CO concentration can thus result in a toxic level of COHb in the blood and lead to a decrease in the amount of oxygen transported by hemoglobin to organs and tissues. Up to 46 % of CO poisoning victims develop delayed neuropsychological sequelae, including cognitive deficits, motor disturbances, and vestibular abnormalities [15]. Recent reports have also highlighted the contribution of CO poisoning to the incidence of cardiac events [16, 17] because the affinity of CO for myoglobin is even greater than it is for Hb [1]. The binding of CO to cardiac myoglobin can cause myocardial depression, hypotension, arrhythmia, and even death [12].

To this day, one of the greatest challenges facing any emergency department in cases of CO poisoning is the need to promptly dissociate COHb to save patients’ lives. However, the treatment options in the emergency physicians’ arsenal remain limited and are often associated with severe side effects. Currently, the widely accepted methods of management, NBO therapy and HBO therapy, maximize the PaO_2_ level, thereby increasing O_2_Hb and indirectly dissociating CO from COHb. NBO treatment involves removing the patient from the source of exposure and administering 100 % NBO for 4 to 6 h to remove over 90 % of the CO until the COHb concentration is less than 5 %. This regimen brings the half-life of COHb down to 74 min, as opposed to 320 min when breathing air [18]. The administration of oxygen speeds the elimination of CO from the body. Without oxygen therapy, the elimination half-life of CO is 4 to 5 h. Supplementation with 100 % oxygen via a tight-fitting mask at NBO pressure cuts the elimination half-life by approximately 50 %, whereas the use of HBO, i.e., the use of pure oxygen to speed and enhance the body’s natural ability to heal, at 2.5 atm decreases the elimination half-life to 20 min [18, 19]. However, HBO should be administered within 6 h of exposure [7] for a duration of about 9 h [20], and NBO should be administered when CO poisoning is suspected and before laboratory confirmation. For decades, HBO has been used for severe CO poisoning, but its indication remains controversial [21–25] because of the action of O_2_, which can result in oxygen toxicity seizures and barotrauma, including pneumothorax and hemorrhage. Furthermore, there is no real evidence that HBO administration for CO poisoning reduces the incidence of adverse neurologic outcomes [26].

Additionally, because of the equipment required and its inconvenience, HBO therapy is often not a practical option. HBO therapy requires special equipment, and the institutions amenable to setting up a hyperbaric chamber are limited. Currently in Japan, according to the 2014 Japanese Society of Hyperbaric and Undersea Medicine registry, there are only 591 such facilities nationwide [27]. Phototherapy, i.e., the use of visible light to dissociate COHb, appears to be an appealing and viable alternative for addressing CO poisoning. Unlike NBO and HBO therapies, light irradiation therapy works directly to dissociate CO from COHb; it does not increase the PaO_2_, is not known to result in any complications, and appears to be safe.

The phototherapy method was originally developed from an early hypothesis that light irradiation induces the photodissociation of O_2_Hb. This hypothesis centers on the fact that O_2_Hb absorbs the light radiation, and at a probability of approximately 10 %, the photodissociation will release the O_2_ molecule and restore hemoglobin [28]. Extrapolating this finding to COHb, and despite the similarities in the absorption spectra of O_2_Hb and COHb, with the significant difference (>10-fold) in the quantum yields of photodissociation (which is 98 % for COHb), it becomes possible to achieve selective decomposition of COHb in the bloodstream with a minimum effect on the O_2_Hb. The typical absorption spectra of O_2_Hb, COHb, and reduced O_2_Hb, i.e., hemoglobin (Hb), in vitro are shown in Fig. 3. Light irradiation dissociates CO from the COHb complex and, with a constant O_2_ supply, might re-establish the physiological O_2_Hb bond. Irradiation of a COHb sample by light at various frequencies has been documented to split the CO-Hb bond and presumably restore O_2_Hb binding. In a landmark study, Kashimura et al. [29] infused rats with synthetic artificial oxygen-carrying hemoglobin vesicles (HbV) and exposed the rats to light of 500,000 lux and showed that the COHb significantly dissociated (26.1 ± 2.4 %) after 90 min of exposure.

We further explored the theory and experimental success by examining the rate of dissociation achieved with light emitted at different illuminances (0, 100,000, and 500,000 lux) in human in vitro blood samples highly saturated with COHb. Results of the light irradiation were promising, with a relatively high dissociation rate achieved with exposure to 500,000 lux within a short (15-min) period.

In the in vitro study described herein, the Hb dissociated from the COHb complex presumably bound with O_2_ and formed an O_2_Hb molecule, and the remaining CO molecule most likely volatilized in air. In the human body, photodecomposition of COHb and CO removal will be most effective in the lungs and skin. However, the unbound CO has a strong propensity for attachment to Hb, and to prevent such de novo COHb complex formation, sufficient O_2_ is required even in instances of low COHb dissociation.

Our application of light irradiation to in vitro human blood samples yielded encouraging data, and we consider this a first step toward clinical application of COHb dissociation for CO poisoning patients. The path to clinical application must include both tests in large animal models of CO poisoning and the conceptualization and design of devices, particularly devices that can be used at the scene.

The application of light irradiation to patients with a high COHb concentration will require application of the light as directly and closely as possible to the CO-contaminated blood. The optimal phototherapy effect will be achieved when the light is aimed as close to the oxygen source as possible, namely the lungs. The authors have envisioned a combination of options for the urgent care of CO poisoning patients. One is an extracorporeal light radiation-emitting jacket that could ideally be used to provide COHb dissociation therapy at the scene of trauma, where the patient requires it most. Its easy handling would allow paramedics to place it on the patient during emergency transport to a hospital or trauma center. The second is a light-emitting catheter that, upon patient admission, could be introduced into the pulmonary artery or the right side of the heart to dissociate the COHb complex, thus allowing O2 to bind with Hb while CO is exhaled through the lungs. Development of a light radiation-emitting jacket would need to take into account the optical properties of the human skin for determination of the effective wavelength of the penetrating radiation. This could, however, be achieved by calculating the action spectra of HbCO within the cutaneous layers. Furthermore, the insertion and indwelling of the specifically designed light radiation-emitting catheter would probably require training.

### Limitations

Our study had limitations. The first is the small sample size, and the second is the inherent disadvantage of an in vitro study, despite our use of human blood. Second, the refraction and barrier effect that the light would encounter in cutaneous and subcutaneous tissues of actual patients was not simulated. Third, medical histories, including any underlying chronic disorders, medications, or any prior hemoglobin studies, were not obtained from the volunteers who donated blood.

## Conclusions

We attempted and achieved photodissociation of COHb in in vitro human blood samples by light irradiation. We anticipate performing a larger study to further confirm our results, which have convinced us of the real possibility of readily performed, acute photodissociation therapies for patients with CO poisoning, therapies that are imperative if we wish to meet the challenge of saving the lives of individuals exposed to CO.

## Abbreviations

CO: Carbon monoxide
COHb: Carboxyhemoglobin
HBO: Hyperbaric oxygen
NBO: Normobaric oxygen
O_2_Hb: Oxyhemoglobin
